# SPATA18 Expression Predicts Favorable Clinical Outcome in Colorectal Cancer

**DOI:** 10.3390/ijms23052753

**Published:** 2022-03-02

**Authors:** Akane Sugimura-Nagata, Akira Koshino, Kazuhiro Nagao, Aya Nagano, Masayuki Komura, Akane Ueki, Masahide Ebi, Naotaka Ogasawara, Toyonori Tsuzuki, Kenji Kasai, Satoru Takahashi, Kunio Kasugai, Shingo Inaguma

**Affiliations:** 1 Division of Gastroenterology, Department of Internal Medicine, School of Medicine, Aichi Medical University, Nagakute 480-1195, Japan; nagata.akane.240@mail.aichi-med-u.ac.jp (A.S.-N.); koshino.akira.255@mail.aichi-med-u.ac.jp (A.K.); nagao.kazuhiro.621@mail.aichi-med-u.ac.jp (K.N.); ebi.masahide.814@mail.aichi-med-u.ac.jp (M.E.); ogasawara.naotaka.667@mail.aichi-med-u.ac.jp (N.O.); kasugai.kunio.527@mail.aichi-med-u.ac.jp (K.K.); 2 Department of Experimental Pathology and Tumor Biology, Graduate School of Medical Sciences, Nagoya City University, Nagoya 467-8601, Japan; aya.ngn@med.nagoya-cu.ac.jp (A.N.); komura@med.nagoya-cu.ac.jp (M.K.); c211705@ed.nagoya-cu.ac.jp (A.U.); sattak@med.nagoya-cu.ac.jp (S.T.); 3 Surgical Pathology, School of Medicine, Aichi Medical University, Nagakute 480-1195, Japan; tsuzuki@aichi-med-u.ac.jp; 4 Department of Pathology, School of Medicine, Aichi Medical University, Nagakute 480-1195, Japan; kkasai@aichi-med-u.ac.jp; 5 Department of Pathology, East Medical Center, Nagoya City University, Nagoya 464-8547, Japan

**Keywords:** colorectal cancer (CRC), immunohistochemistry, SPATA18, p53 immunoreactivity, cellular proliferation

## Abstract

Dysregulation of mitochondrial quality control has been reported to be associated with cancer and degenerative diseases. SPATA18 (spermatogenesis-associated 18, also known as Mieap) encodes a p53-inducible protein that can induce lysosome-like organelles within mitochondria that eliminate oxidized mitochondrial proteins and has tumor suppressor functions in mitochondrial quality control. In the present study, 268 primary colorectal cancers (CRCs) were evaluated immunohistochemically for SPATA18 expression to assess its predictive utility and its association with cellular proliferation activity. Furthermore, the association with p53 immunoreactivity, a surrogate marker for *TP53* mutation, was analyzed. Non-neoplastic colonic mucosa showed cytoplasmic SPATA18 expression. Seventy-two percent of the lesions (193/268) displayed high SPATA18 expression in the cytoplasm of CRC cells. Univariate analyses revealed significant associations between SPATA18 expression and tumor size (*p* < 0.0001), histological differentiation (*p* = 0.0017), and lymph node metastasis (*p* = 0.00039). The log-rank test revealed that patients with SPATA18-high CRCs had significantly better survival than SPATA18-low patients (*p* < 0.0001). Multivariate Cox hazards regression analysis identified tubular-forming histology (hazard ratio [HR] = 0.25), age < 70 years (HR = 0.50), and SPATA18-high (HR = 0.55) as potential favorable factors. Lymph node metastasis (HR = 1.98) and peritoneal metastasis (HR = 5.45) were cited as potential independent risk factors. Cellular proliferation activity was significantly higher in SPATA18-high tumors. However, no significant correlation was detected between SPATA18 expression and p53 immunoreactivity or *KRAS*/*BRAF* mutation status. On the basis of our observations, SPATA18 immunohistochemistry can be used in the prognostication of CRC patients.

## 1. Introduction

Multiple biomarkers have been identified to assist in disease diagnosis and to predict treatment efficacy and patient outcomes for cancers such as colorectal cancer (CRC) [1]. Recently, our group established a tissue microarray-based cohort to explore prognostic markers, successfully identifying several prognostic markers in CRC patients [2,3,4,5,6].

Mitochondria are maternally inherited, cytoplasmic organelles originating from symbiotic bacteria and are indispensable for bioenergy, biosynthesis, and signaling for stress-sensing for adaptations to the environment [7,8]. Mitochondria quality control, which prevents the accumulation of defective mitochondria, is indispensable for cell homeostasis. Dysregulation of mitochondrial quality control has been reported to be associated with cancer and degenerative diseases [7,8]. SPATA18 (spermatogenesis-associated 18, also known as Mieap) encodes a p53-inducible protein that has been reported to be able to induce lysosome-like organelles within mitochondria that eliminate oxidized mitochondrial proteins and is considered to contribute as a tumor suppressor through mitochondrial quality control. [9] On the basis of Spata18-deficient mice models, tumor suppressive effects of SPATA18 were suggested; inactivation of SPATA18-regulated mitochondrial quality control leads to accumulation of unhealthy mitochondria and increased mitochondrial ROS (reactive oxygen species) generation, which probably promotes cancer development and aggressiveness [9,10].

The tumor suppressor p53 was first identified in 1979 as an oncoprotein [11,12,13]. On the basis of the evidence that *TP53* is one of the most mutated genes in human cancers, numerous studies including gene mutation analyses have been performed to clarify the function of p53. The essential functions of p53 are considered to be as follows: (1) p53 is a transcription factor that activates its target genes by binding to specific sequences [14]; and (2) p53 harbors tumor suppressive effects through cell cycle arrest, apoptosis, DNA repair, and anti-angiogenesis [15]. However, based on murine models, it was considered that cell cycle arrest and apoptosis are not required for tumor suppression [16,17]. (3) p53 is frequently mutated in a broad range of human cancers [18], and mutations in *TP53* result in different isoforms with variable transcriptional activity, which leads to different cancer phenotypes [19].

p53 immunohistochemistry is now considered as an accurate surrogate marker reflecting the underlying *TP53* mutational status frequently used in tumor diagnostics [20]. It has long been recognized that nonsynonymous *TP53* missense mutations result in nuclear accumulation of p53 protein, which can be detected as overexpression in the form of diffuse strong nuclear positivity involving at least 80% of the tumor cells but usually almost 100% [20]. To date, other abnormal p53 expression patterns, cytoplasmic expression [21] and complete loss [22], have been recognized as correlating with the presence of a *TP53* mutation.

The present study examined the expression status of SPATA18 in CRCs. The associations of SPATA18 expression with clinicopathological features and clinical outcomes were analyzed to assess their potential for clinical use. In addition, the associations between SPATA18 and cellular proliferation markers or p53 immunoreactivity were analyzed to characterize SPATA18-expressing CRCs.

## 2. Results

### 2.1. Expression of SPATA18 in Non-Neoplastic Colonic Mucosa and CRCs

**Figure 1:**
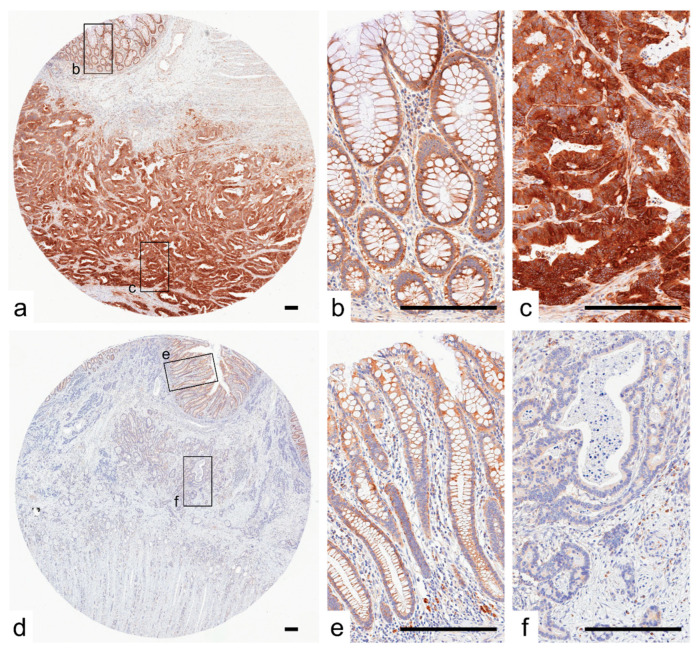
Representative images of SPATA18 immunostaining. (**a**–**c**), Representative images of SPATA18-high CRC. SPATA18 was weakly and strongly expressed in the cytoplasm of non-neoplastic colonic epithelial cells (**b**) and CRC cells (**c**), respectively. (**d**–**f**), Representative images of SPATA18-low tumors. CRC cells exhibited lower levels of cytoplasmic SPATA18 expression (**f**) than non-neoplastic colonic epithelial cells (**e**). SPATA18-positive immune cells were present in both non-neoplastic and neoplastic stroma. Bar, 200 μm.

**Figure 2:**
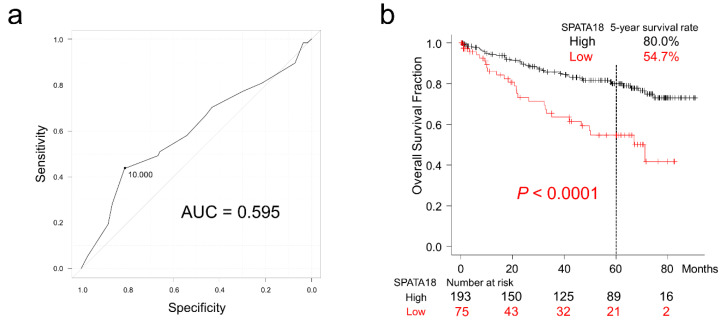
Overall survival of patients with colorectal cancer classified according to SPATA18 expression. (**a**), ROC curve for SPATA18 expression at the patient’s death. The cut-off value was defined from the closest point to the upper-left side. (**b**), Kaplan-Meier curves for patients classified by SPATA18 expression.

Representative images of CRC cases with or without SPATA18 expression are presented in Figure 1. In non-neoplastic colonic mucosa, SPATA18 was weakly expressed in the cytoplasm of colonic epithelial cells. In addition, SPATA18-positive inflammatory cells were observed (Figure 1b,e). The cutoff value for SPATA18 immunohistochemistry was defined as 10% from the ROC (Receiver Operating Characteristic) curves for patient survival at 5-years (Figure 2a). In total, 28% of the lesions (75/269) exhibited lower SPATA18 expression in CRC cells (Table 1).

The clinical, pathological, and immunohistochemical features of the analyzed tumors are summarized in Table 1 according to SPATA18 expression. SPATA18 expression was associated with tumor size (*p* < 0.0001), histological differentiation (*p* = 0.0017), and lymph node metastasis (*p* = 0.00039).

### 2.2. Survival Analyses of Patients with CRC

The cut-off value for SPATA18 expression was determined at 10% by the ROC curve for patient survival at 5 years and the area under curve (AUC) was 0.595 (95% confidence interval [CI] = 0.50–0.69; Figure 2). Patients with SPATA18-low CRC had significantly worse 5-year survival (54.7% vs. 80.0%; *p* < 0.0001; Figure 2b). Analysis of data from TCGA (The Cancer Genome Atlas) also revealed unfavorable survival in CRC patients with *SPATA18*-low tumors (Supplementary Figure S1). Multivariate Cox hazards regression analysis identified tubular-forming histology (hazard ratio [HR] = 0.25, 95% confidence interval [CI] = 0.13–0.47, *p* < 0.0001), younger age (<70 years old, HR = 0.50, 95% CI = 0.29–0.86, *p* = 0.012), and high tumor SPATA18 expression (HR = 0.55, 95% CI = 0.32–0.94, *p* = 0.029), as potential favorable factors. The analysis also revealed the presence of lymph node metastasis (HR = 1.98, 95% CI = 1.14–3.45, *p* = 0.015), and peritoneal metastasis (HR = 5.45; 95% CI = 3.05–9.73, *p* < 0.0001) as potential independent risk factors for patients with CRC (Table 2).

Among the analyzed tumors by using Kaplan-Meier Plotter pan-cancer RNA-seq data, papillary renal cell carcinoma, thyroid carcinoma, endometrial carcinoma, clear cell renal cell carcinoma, HER2 type breast cancer, basal type breast cancer, sarcoma, lung adenocarcinoma, and head and neck squamous cell carcinoma showed lower risk in *SPATA18*-expressing tumors (HR = 0.22–0.72; Table 3). In contrast, bladder carcinoma showed higher risk in *SPATA18*-expressing tumors (HR = 1.43; Table 3).

### 2.3. SPATA18 Was Correlated with Cellular Proliferation Markers

**Figure 3:**
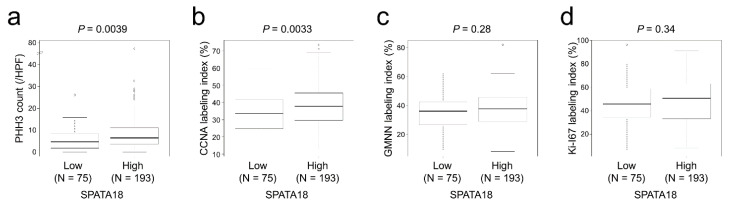
Cellular proliferation marker expression classified according to SPATA18 expression. (**a**), SPATA18-high tumors contained a significantly higher number of PHH3-positive cells. (**b**), CCNA labeling index was significantly higher in SPATA18-high tumors. (**c**,**d**), No significant difference was detected in GMNN or Ki-67 labeling indices in SPATA18-high and -low tumors.

Immunohistochemical staining analyses revealed that SPATA18-high tumors contained a significantly higher number of PHH3 (phospho-histone H3)-positive cells (*p* = 0.0039; Figure 3a). The CCNA (cyclin A) labeling index was significantly higher in SPATA18-high tumors (*p* = 0.0033; Figure 3b). In contrast, no significant difference was detected between SPATA18 expression and GMNN (geminin) or Ki-67 labeling indices.

### 2.4. SPATA18 Showed No Correlation with p53 Immunoreactivity or KRAS/BRAF Mutations

**Figure 4:**
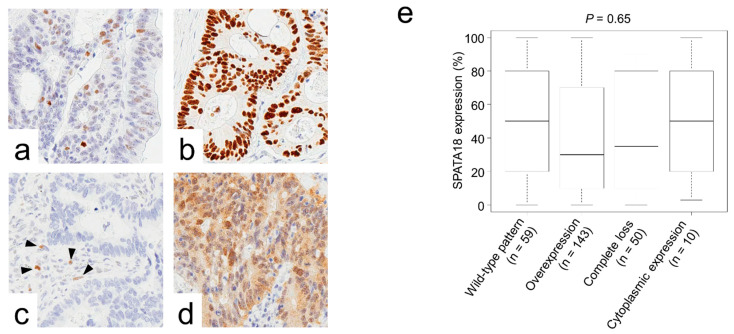
Association of p53 immunoreactivity and SPATA18 expression. (**a**–**d**), Representative images of p53 immunoreactivity. (**a**) Wild-type pattern, (**b**) overexpression, (**c**) complete loss, and (**d**) cytoplasmic expression. Arrow heads indicate internal controls. (**e**), No significant correlation was detected between SPATA18 expression and p53 expression patterns.

Representative images of CRC cases with various p53 immunoreactivity patterns are presented in Figure 4a–d. No significant correlation was detected between SPATA18 expression and p53 expression patterns (Figure 4e).

No correlation was found between SPATA expression and *KRAS*/*BRAF* mutations. (Supplementary Figure S2).

## 3. Discussion

Dysregulation of mitochondrial quality control has been reported to be associated with cancer and degenerative diseases [7,8]. SPATA18, a p53-inducible protein, has been reported to be involved in mitochondrial quality control [10]. When *Spata18* knock out mice were crossed with *Apc*^Min/+^ mice, which are known to develop multiple benign tumors in the small intestine [23,24], *Spata18*-deficient *Apc*^Min/+^ mice showed a much shorter lifespan with a higher number and size of intestinal tumors compared with *Spata18*-WT *Apc*^Min/+^ mice [9]. Moreover, intestinal tumors in *Spata18*-deficient *Apc*^Min/+^ mice showed more advanced grades of adenomas and adenocarcinomas than *Spata18*-WT *Apc*^Min/+^ mice [9]. These results suggested the tumor suppressive effects of *Spata18* during intestinal adenocarcinoma development; inactivation of Spata18-regulated mitochondrial quality control leads to accumulation of unhealthy mitochondria and increased mitochondrial ROS generation, which probably promotes cancer development and aggressiveness [9]. Furthermore, using web-based programs, lower hazard ratios were identified in carcinomas arising in the kidney, thyroid, uterus, and breast (Table 3). In contrast, bladder carcinomas indicated a higher risk in *SPATA18*-high tumors with unknown mechanisms.

Mitochondria are extremely dynamic, and dynamin-related protein-1 (Drp1)-regulated balance of fission and fusion dictates their morphology. Altered mitochondrial dynamics are a critical feature of *KRAS*-dependent cellular transformation; oncogenic *KRAS* stimulates mitochondrial fragmentation via ERK1/2-mediated phosphorylation of Drp1 [25,26]. Additionally, remodeling of the mitochondrial network upon oncogenic *KRAS* expression has been reported to affect increased ROS generation [26]. On the basis of these notions, we analyzed the gene mutation status and SPATA18 expression in CRCs; however, no correlation was detected (Supplementary Figure S2).

The prognostic impacts of cellular proliferation markers have been controversial in CRC patients [27,28,29,30,31,32]. Recently, our group revealed that higher expression of PHH3, which is expressed in late G2 and M phases, is associated with lower pT stage and favorable clinical outcome in CRC patients [4]. These observations were partly explained by our additional in vitro study in which PBK (also known as TOPK) was demonstrated to accelerate cellular proliferation with direct phosphorylation of HH3 (histone H3) along with the suppression CRC cell migration and invasion [33]. In the present study, SPATA18-high CRCs showed higher PHH3 counts and CCNA labeling indexes than SPATA18-low tumors with favorable clinical outcomes. These results are in line with our previous results that CRCs with proliferating cells show favorable clinical outcomes [4].

SPATA18 has been reported as a p53-inducible protein; however, its expression has been demonstrated to be regulated in part by gene promoter methylation [34]. In primary CRCs, it was reported that the methylation of *SPATA18* promoters was observed in only 5 out of 57 patients (9%) [34]. Furthermore, with frequent *TP53* mutations in nearly 50% of CRCs, we hypothesized that dysregulated p53 transcriptional activity leads to abnormal expression of SPATA18. To assess this, we analyzed the association between SPATA18 expression and p53 immunoreactivity, an established surrogate marker for *TP53* mutation; however, no significant correlation was found. This may be because mutations in *TP53* result in different isoforms with variable transcriptional activity, which leads to different cancer phenotypes [19].

Serum carcinoembryonic antigen (CEA) is one of the established biomarkers for diagnosis, monitoring the recurrence and metastasis and the evaluation of chemotherapy in CRC [35,36]. Recently, the prognostic impacts of CEA have been evaluated by using ROC curves and reported that post-operative CEA (AUC = 0.686, 95% CI = 0.657–0.714) was better than pre-operative CEA (AUC = 0.621, 95% CI = 0.592–0.650) in stage II CRC [35]. In the present study, the ROC curve revealed that AUC of SPATA18 was 0.595 (95% CI = 0.50–0.69; Figure 2a). These differences may be due to the characteristics of the markers, evaluation methods, and/or cohorts (e.g., patient number and stage): CEA is aberrantly expressed by neoplastic cells and detected by serum chemiluminescent immunoassay; the decreased or lost expression of SPATA18 in tumor cells was evaluated by immunohistochemistry.

The present study immunohistochemically evaluated the expression of SPATA18 in CRCs. SPATA18-high status was identified as a potential favorable factor for CRC patients. SPATA18-high tumors contained a significantly higher number of PHH3-positive cells. However, no significant correlation was detected between SPATA18 expression and p53 immunoreactivity or *KRAS*/*BRAF* mutation status. According to our observations, SPATA18 immunohistochemistry can be used in the prognostication of CRC patients. ROS inhibitors or anti-oxidants may be applied to cases with lower SPATA18 tumors or oncogenic *KRAS*-positive CRCs.

## 4. Materials and Methods

### 4.1. Tissue Samples

The Institutional Ethical Review Board of Aichi Medical University Hospital approved this project without the need for patient consent by giving them the opportunity to opt out. Two hundred and sixty-nine formalin-fixed, paraffin-embedded (FFPE) samples of primary colorectal tumors resected at Aichi Medical University Hospital from 2009 to 2012 were collected depending on the availability of tissue samples and clinical information. After surgery, patients were followed for up to 90 months. All tumors were diagnosed as invasive and naïve to chemotherapy or radiotherapy according to TNM classification [37]. Tumors with glandular formation (>50%) or mucus production (>50% of the area) were defined as having a differentiated or mucus-producing histology. A single 4.5-mm core tumor tissue sample derived from an FFPE specimen was assembled into multitumor blocks containing up to 30 samples. All cores were obtained from invasive areas, and approximately 20% of cores contained an invasive front. Non-neoplastic colonic mucosae adjacent to the tumor were also immunohistochemically analyzed.

### 4.2. Immunohistochemistry

The antibodies used in the present study are summarized in Supplementary Table S1. Immunohistochemistry was performed using a Leica Bond-Max (Leica Biosystems, Wetzlar, Germany) or Ventana BenchMark XT automated immunostainer (Roche Diagnostics, Basel, Switzerland). Signals were visualized using 3,3′-diaminobenzidine. SPATA18 expression was independently evaluated by two researchers (AS-N and SI). The concordance rates of the initial immunohistochemical evaluation are presented in Supplementary Table S2. The results of discordant cases were confirmed via discussion.

The data for cellular proliferation markers were cited from our previous study [4]. In brief, Ki-67, CCNA, and GMNN labeling indices were determined by counting >500 tumor cells per case in a high-power field (×400). The number of PHH3-positive cells was counted under the same magnification.

p53 immunoreactivity was classified as follows: wild-type, overexpression, complete loss, and cytoplasmic expression [20]. In the evaluation of complete loss of p53 expression, cases without internal controls such as fibroblasts and lymphoid cells were eliminated from the study.

### 4.3. Statistical Analyses

Statistical analyses were performed using EZR software version 1.41 [38]. The cutoffs for immunohistochemistry were defined as the value closest to the upper-left corner in the ROC curves for patient survival at 5 years. The chi-squared test, Fisher’s exact test, Cochran–Armitage trend test, Mann-Whitney U test, or the Kruskal-Wallis test was performed to analyze the statistical correlation between categorical data. Simple Bonferroni correction for multiple hypothesis testing was applied for adjustment at a two-sided alpha level of 0.0042 (=0.05/12).

For survival analyses, Kaplan–Meier survival estimates were calculated with the log-rank test. Cox proportional hazards regression analysis was performed to analyze the associations of survival with other factors. The initial model included the following variables: sex (male vs female), age (<70 years old vs. ≥70 years old), tumor size (<5 cm vs. ≥5 cm), primary tumor location (right-sided colon vs left-sided colon vs rectum), pT stage (pT2 vs. pT3 vs. pT4), tumor histology (moderate to well-differentiated vs. poorly differentiated), mucus production (positive vs. negative), lymph node metastasis (positive vs. negative), peritoneal metastasis (positive vs. negative), distant organ metastasis (positive vs negative), surgical status (complete vs. incomplete resection), mismatch repair system status (preserved vs. deficient), and immunohistochemical data (SPATA18-high vs. SPATA18-low). A backward elimination with a threshold of *p* < 0.05 was used to select variables in the final model.

### 4.4. Survival Analyses Using Web Site Programs

Data from TCGA were analyzed using the UCSC Xena program (https://xena.ucsc.edu/ (accessed on 31 January 2022)). The best cut-off values were automatically set by the program for each tumor type.

Survival analyses were performed using Kaplan-Meier Plotter pan-cancer RNA-seq data according to SPATA18 expression (https://kmplot.com/analysis/ (accessed on 31 January 2022)). The best cut-off values were automatically set by the program for each tumor type.

### 4.5. Gene Mutation Analyses

*KRAS* mutation status was collected from the medical records. *BRAF* V600E mutation analyses were performed by polymerase chain reaction (PCR)-direct sequencing using the following primers: *BRAF* forward, tgc ttg ctc tga tag gaa aat g; *BRAF* reverse, cag ggc caa aaa ttt aat cag t.

## Figures and Tables

**Table 1:** Characteristics of colorectal carcinomas classified according to SPATA18 expression.

			SPATA18					
	Total No.		High		Low		*p*-Value	
	268	(100%)	193	(72%)	75	(28%)		
Sex								a
Male	143	[53%]	106	[55%]	37	[49%]	0.49	
Female	125	[47%]	87	[45%]	38	[51%]		
Age, years (mean ± S.D.)	68.6 ± 12.6		68.3 ± 12.3		69.1 ± 13.3		0.63	b
Size, cm (mean ± S.D.)	5.0 ± 2.6		4.62 ± 2.29		5.98 ± 2.96		<0.0001	b
Tumor location								a
Right-sided colon	123	[46%]	87	[45%]	36	[48%]	0.91	
Left-sided colon	86	[32%]	63	[33%]	23	[31%]		
Rectum	59	[22%]	43	[22%]	16	[21%]		
pT stage								c
pT2	36	[13%]	32	[17%]	4	[6%]	0.036	
pT3	188	[70%]	136	[70%]	52	[69%]		
pT4	44	[16%]	25	[13%]	19	[25%]		
Histological differentiation								a
Well to moderately	241	[90%]	181	[94%]	60	[80%]	0.0017	
Poorly	27	[10%]	12	[6%]	15	[20%]		
Mucus production								d
Positive	14	[5%]	11	[6%]	3	[4%]	0.76	
Negative	254	[95%]	182	[94%]	72	[96%]		
Lymph node metastasis								a
Positive	99	[39%]	77	[42%]	22	[32%]	0.00039	
Negative	153	[61%]	106	[58%]	47	[68%]		
Peritoneal metastasis								a
Positive	50	[19%]	33	[17%]	17	[23%]	0.38	
Negative	218	[81%]	160	[83%]	58	[77%]		
Distant organ metastasis								a
Positive	44	[16%]	30	[16%]	14	[19%]	0.66	
Negative	224	[84%]	163	[84%]	61	[81%]		
Operation status								a
Complete resection	236	[88%]	175	[91%]	61	[81%]	0.057	
Incomplete resection	32	[12%]	18	[9%]	14	[19%]		
MMR system status								a
Deficient	238	[88%]	173	[90%]	65	[87%]	0.63	
Preserved	30	[12%]	20	[10%]	10	[13%]		

a, *p*-values were calculated by the chi-squared test for SPATA18 expression. b, *t*-test, c, Cochran-Armitage trend, or d, Fisher’s exact test was used to calculate *p*-values. The Bonferroni-corrected *p*-value for significance was *p* ≈ 0.0042 (0.05/12).

**Table 2:** Multivariable Cox hazards analysis of colorectal cancer patients.

	Hazard	95% CI		
	Ratio	Min	Max	*p*-Value
Well to moderately differentiated histology	0.25	0.13	0.47	<0.0001
Age (<70)	0.50	0.29	0.86	0.012
SPATA18 high	0.55	0.32	0.94	0.029
Lymph node metastasis	1.98	1.14	3.45	0.015
Peritoneal metastasis	5.45	3.05	9.73	<0.0001

The multivariable Cox hazards analysis model initially included sex, age, primary tumor location, tumor size, pT stage, operation status, tumor histology, mucus production, lymph node metastasis, distant organ metastasis, peritoneal metastasis, mismatch repair system status, and immunohistochemistry for SPATA18. A backward elimination with a threshold of *p* = 0.05 was used to select variables in the final model.

**Table 3:** Survival analyses in Kaplan–Meier Plotter according to *SPATA18* expression.

	Patient	Hazard	95% CI		Log-Rank
	No.	Ratio	Min	Max	*p*-Value
Papillary renal cell carcinoma	287	0.22	0.12	0.40	<0.0001
Thyroid carcinoma	502	0.29	0.10	0.79	0.01
Endometrial carcinoma	542	0.35	0.22	0.56	<0.0001
Clear cell renal cell carcinoma	530	0.36	0.26	0.48	<0.0001
HER2 type breast cancer	295	0.51	0.26	1.00	0.047
Basal type breast cancer	309	0.59	0.36	0.98	0.04
Sarcoma	259	0.63	0.42	0.94	0.022
Lung adenocarcinoma	504	0.67	0.50	0.91	0.0084
Head-neck squamous cell carcinoma	499	0.72	0.55	0.94	0.015
Bladder carcinoma	404	1.43	1.03	1.98	0.031

RNA-seq data were analyzed using the Kaplan-Meier Plotter program. Note that no significant difference was detected in cervical squamous cell carcinoma, esophageal adenocarcinoma, esophageal squamous cell carcinoma, hepatocellular carcinoma, luminal A and B type breast cancer, lung squamous cell carcinoma, normal type breast cancer, ovarian cancer, pancreatic ductal adenocarcinoma, stomach adenocarcinoma, and testicular germ cell tumor.

## Data Availability

The datasets used and/or analyzed during the present study are available from the corresponding author on reasonable request.

## Supplementary Materials

The following are available online at https://www.mdpi.com/article/10.3390/ijms23052753/s1.
