# Supplementary material for: SPATA18 Expression Predicts Favorable Clinical Outcome in Colorectal Cancer
Source: Int J Mol Sci. 2022 Mar 2;23(5):2753. doi: 10.3390/ijms23052753 (PMC8910917; doi:10.3390/ijms23052753)
Supplement: Supplementary file 1 [file ijms-23-02753-s001.zip › ijms-1613637-supplementary.pdf]

International Journal of
Molecular Sciences

MDPI

Supplementary materials: SPATA18 expression predicts favorable clinical outcome in colorectal cancer

Akane Sugimura-Nagata, Akira Koshino, Kazuhiro **Nagao, Aya Nagano, Masayuki Komura, Akane Ueki, Masahide Ebi, Naotaka Ogasawara, Toyonori Tsuzuki, Kenji Kasai, Satoru Takahashi, Kunio Kasugai, and Shingo Inaguma**

Supplementary Table S1. Antibodies and conditions for immunohistochemistry

Genes	Reagent	Dilution	Antibodies
CCNA	IV	100	sc-751, Santa Cruz Biothechnology, Inc. (Dallas, TX)
GMNN	OV	500	EPR14637, Abcam (Cambridge, UK)
Ki-67	OV	100	Clone MIB-1, Dako/Agilent, (Santa Clara, CA)
MLH1	OV	200	Clone G168-728, BD Biosciences, (Franklin Lakes, NJ)
MSH2	OV	200	Clone G219-1129, BD Biosciences, (Franklin Lakes, NJ)
MSH6	OV	400	Clone 44/MSH6, BD Biosciences, (Franklin Lakes, NJ)
SPATA18	BM	1,000	EPR13704, Abcam (Cambridge, UK)
TP53	BM	500	Clone DO7, Leica Biosystems (Wetzlar, Germany)
PHH3	BM	500	Cell Marque™, Millipore SIGMA, (Rocklin, CA)
PMS2	OV+Linker	50	Clone A16-4, BD Biosciences, (Franklin Lakes, NJ)

BM, Leica BondMax; IV, Ventana iView reagent; OV, Ventana OptiView reagent. Antigen retrieval was performed with heat activation in high pH buffer.

Supplementary Table S2. Concordance rate of initial immunostaining evaluation

Immunohistochemistry	Concordance Rate (%)	k coefficient	95% Confidence Interval	
			Min	Max
SPATA18	89.9	0.75	0.67	0.84

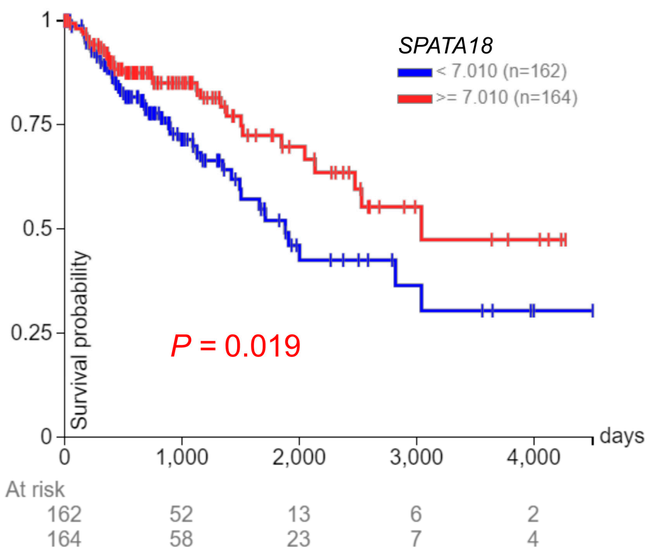

Supplementary Figure S1. Overall survival of CRC cases according to *SPATA18* expression. Kaplan-Meier curves for the patients grouped according to *SPATA18* expression levels. Note that patients with CRCs expressing *SPATA18* at lower levels showed significantly worse overall survival.

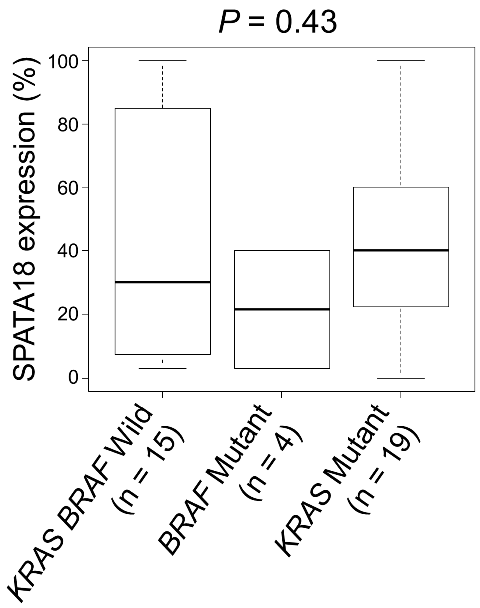

Supplementary Figure S2. SPATA18 expression in the tumors with or without gene mutation. Note that no significant correlation was detected between SPATA18 expression and *KRAS* or *BRAF* mutations.
